# High throughput analysis of B cell dynamics and neutralizing antibody development during immunization with a novel clade C HIV-1 envelope

**DOI:** 10.1371/journal.ppat.1011717

**Published:** 2023-10-25

**Authors:** Rohini Mopuri, Sarah Welbourn, Tysheena Charles, Pooja Ralli-Jain, David Rosales, Samantha Burton, Areeb Aftab, Kirti Karunakaran, Kathryn Pellegrini, William Kilembe, Etienne Karita, Sandrasegaram Gnanakaran, Amit A. Upadhyay, Steven E. Bosinger, Cynthia A. Derdeyn

**Affiliations:** 1 Emory National Primate Research Center, Emory University, Atlanta, Georgia, United States of America; 2 Department of Laboratory Medicine and Pathology, University of Washington, Seattle, Washington, United States of America; 3 Center for Family Health Research in Zambia, Lusaka, Zambia; 4 Center for Family Health Research, Kigali, Rwanda; 5 Theoretical Division, Los Alamos National Laboratory, Los Alamos, New Mexico, United States of America; 6 Department of Pathology and Laboratory Medicine, Emory University, Atlanta, Georgia, United States of America; 7 Infectious Diseases and Translational Medicine Unit, Washington National Primate Research Center, University of Washington, Seattle, Washington, United States of America; National Institute for Communicable Diseases, SOUTH AFRICA

## Abstract

A protective HIV-1 vaccine has been hampered by a limited understanding of how B cells acquire neutralizing activity. Our previous vaccines expressing two different HIV-1 envelopes elicited robust antigen specific serum IgG titers in 20 rhesus macaques; yet serum from only two animals neutralized the autologous virus. Here, we used high throughput immunoglobulin receptor and single cell RNA sequencing to characterize the overall expansion, recall, and maturation of antigen specific B cells longitudinally over 90 weeks. Diversification and expansion of many B cell clonotypes occurred broadly in the absence of serum neutralization. However, in one animal that developed neutralization, two neutralizing B cell clonotypes arose from the same immunoglobulin germline and were tracked longitudinally. Early antibody variants with high identity to germline neutralized the autologous virus while later variants acquired somatic hypermutation and increased neutralization potency. The early engagement of precursors capable of neutralization with little to no SHM followed by prolonged affinity maturation allowed the two neutralizing lineages to successfully persist despite many other antigen specific B cells. The findings provide new insight into B cells responding to HIV-1 envelope during heterologous prime and boost immunization in rhesus macaques and the development of selected autologous neutralizing antibody lineages.

## Author summary

Protection from HIV-1 infection will likely require a vaccine to elicit neutralizing antibody responses that can recognize many different viral strains. However, development of these types of antibodies requires many years of infection and is a continued challenge for vaccine design. Furthermore, most antibodies elicited against HIV-1 envelope (Env) lack neutralizing activity or can only neutralize the matching viral strain. Here we conducted a high throughput longitudinal investigation of how B cells responded to heterologous prime-boost vaccines expressing different HIV-1 Env immunogens when administered to nonhuman primates. In most animals, many B cells recognized the antigen, expanded, evolved through mutation, and persisted during vaccination but without acquiring neutralizing activity. In one animal that developed neutralizing antibodies against the vaccine strain, we traced the evolution of the B cells producing those antibodies over time. We found that two neutralizing antibody-producing B cell families were elicited early in vaccination and gained neutralization strength through expansion, continued mutation, and persistence over time, while also competing with many other non-neutralizing B cells. Our results suggest that early elicitation may be one factor that favors the induction of neutralizing antibodies against HIV Env.

## Introduction

Most persons living with HIV-1 infection develop strain specific (autologous) neutralizing antibody responses and a small subset of these go on to acquire heterologous neutralization breadth after several years of infection [1]. These broadly neutralizing antibodies (bnAbs) from chronic infection have become a singular focus of many vaccination strategies, even though potent and durable strain-specific neutralizing antibodies against globally relevant (tier 2) HIV-1 viruses have only been elicited in humans or nonhuman primate models on a limited basis. As such, promising advances are underway to enhance these responses by prolonging exposure to antigen [2,3]. However, knowledge about the specific B cells that produce neutralizing antibodies in response to vaccination remains limited. Envelope (Env) specific B cells are abundantly elicited by vaccination, but the capability of a single B cell to produce a neutralizing antibody is likely influenced by myriad factors, including the germline features and pairing of the VH and VL chain variable domains [4–7], the lymphoid environment [2], the form of antigen [8] , adjuvant [8–10], and clonal expansion [11,12]. Many studies support that a protective B cell response against HIV-1 will require a vaccine to elicit neutralizing antibodies of high potency, durability, and breadth. To learn more about how Env specific B cell responses develop over time, we evaluated their dynamics in the plasmablast (PB) and circulating memory B cell (MBC) compartments and tracked select neutralizing antibody lineages during and after immunization of rhesus macaque (RM) with DNA, modified vaccinia Ankara (MVA), and Env protein.

In our prior work, we developed vaccines based on two distinct HIV-1 transmitted/founder (T/F) Envs that were associated with either strong or weak autologous and heterologous neutralization responses during infection in humans [11,13]. Because we and others have observed convergent neutralizing antibody responses against HIV-1 T/F Envs in RM and humans [11,14], we used this model to further characterize B cell responses in RM using high throughput methods. We applied immunoglobulin repertoire sequencing of acute PB and RNA sequencing of antigen specific MBC at longitudinal time points to explore their dynamics during and after the immunization series. We also characterized the engagement, expansion, and maturation of two clonotypes with neutralizing activity that emerged in parallel in one animal. The results demonstrate that clonotype diversity, expansion, and SHM levels were not different based on which Env was included in the vaccine or whether neutralizing activity developed. Effective recall of B cell clonotypes by the sequential vaccine components was observed, with persistence of some clonotypes throughout the entire immunization and beyond. Clonal expansion, SHM, recall, and persistence were not sufficient for neutralizing antibodies to develop in most immunized animals; however, precursor frequency and efficiency of antigen engagement, as well as inherent features of the two Env immunogens may have influenced the outcome.

## Results

### Characterizing the trajectory of two neutralizing B cell lineages in an immunized RM

**Fig 1:**
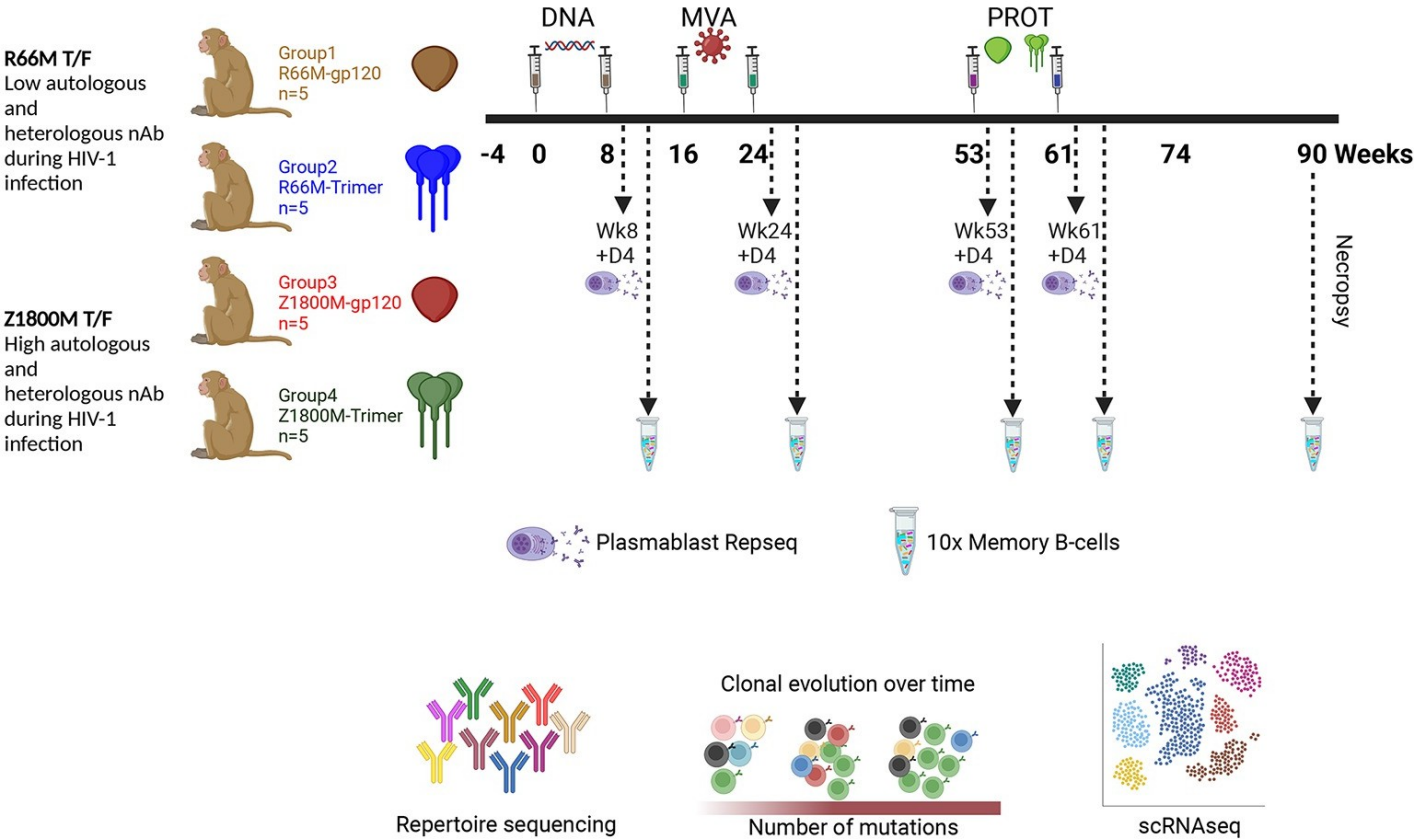
Schematic of immunization regimen and sample collection. An immunization study was conducted previously in RM. The HIV-1 Env immunogens were based on high or low autologous and heterologous neutralization during infection. Samples were collected as follows: Plasmablasts (PB) were isolated 4 days after the second DNA (week 8), second MVA (week 24), first protein (week 53), and second protein (week 61). VH chain bulk RepSeq was performed. PBMC were cryopreserved at 2 weeks after the second DNA (week 8), second MVA (week 24), first protein (week 53), second protein (week 61), and necropsy (week 90). Antigen specific memory B cells (MBC) were sorted using the autologous Env gp120 probe and subjected to VH and VL chain variable domain PCR (and cloning of VDJ region in some cases for mAb expression) or 10X Genomics paired VH and VL chain variable domain sequencing (and synthesis and cloning of VDJ regions for mAb expression in some cases). This figure was created with BioRender.com.

**Fig 2:**
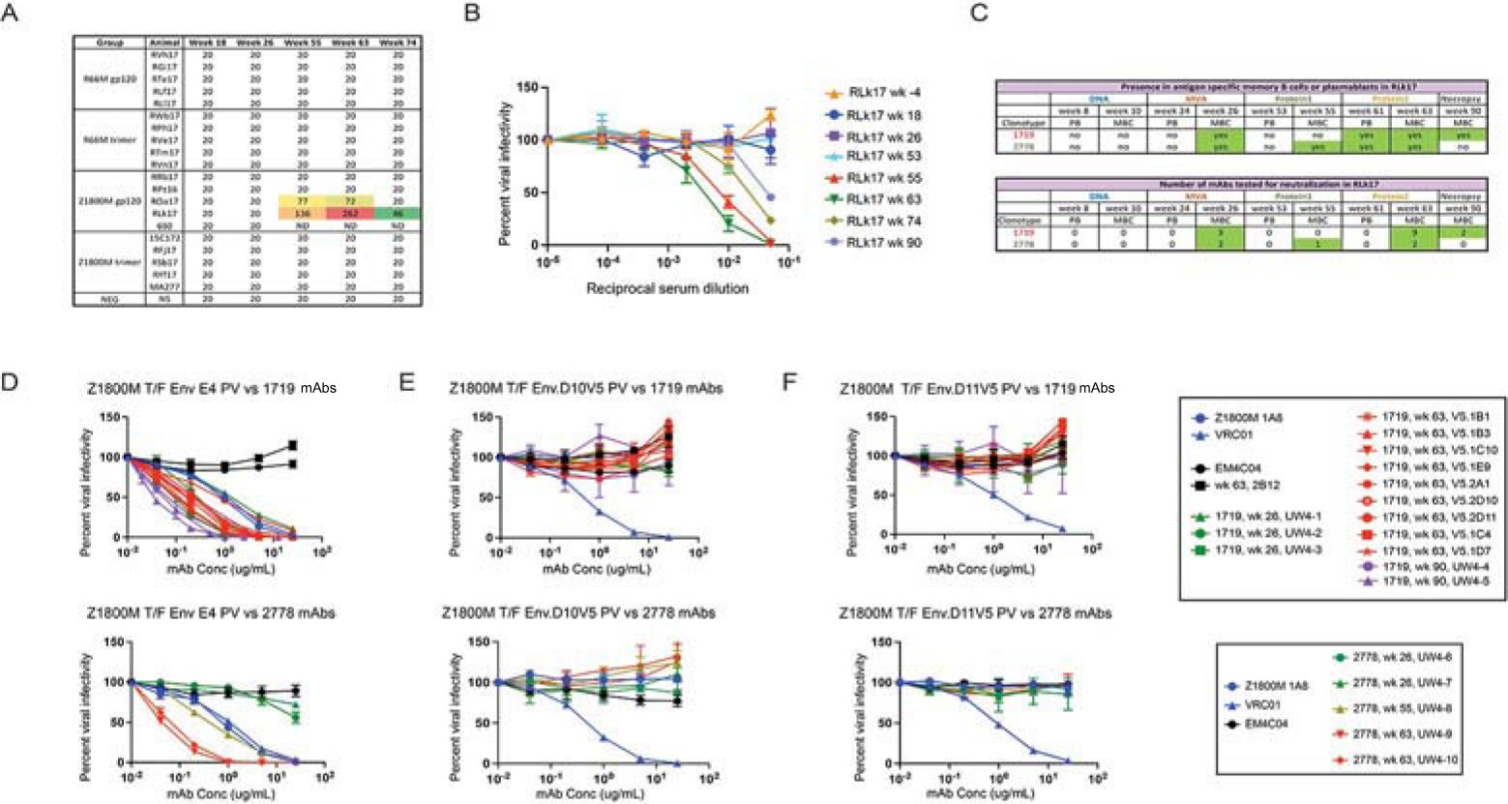
Vaccine elicited neutralizing antibody responses. **A.** Serum neutralizing antibody ID_50_ titers are shown against the autologous Env PV (Z1800M T/F Env or R66M T/F Env) as determined in the TZM-bl assay. Time points measured are week 18, 2 weeks after the first MVA; week 26, 2 weeks after the second MVA; week 55, 2 weeks after the first protein; week 63, 2 weeks after the second protein; week 74, 13 weeks after the second protein. An ID_50_ of 20 indicates that this value could not be calculated using a dilution series that starts at 1:20. ID_50_ values that could be calculated are shown for two animals with warm colors indicated more potent neutralization. **B.** Serum neutralizing activity dose inhibition curves are shown for RM RLk17 at all times included in panel A, along with baseline (week -4), day of first protein immunization (week 53), and necropsy (week 90, 29 weeks after the second protein). The reciprocal of the serum dilution is shown on a log10 scale and plotted against percent viral infectivity. RLk17 neutralization was first detected after the first protein (week 55), increased after the second protein (week 63), and waned over time once immunization ceased. Note that serum neutralization was not detected at week 26. **C.** The table shows at what time points the RLk17 B cell clonotypes with neutralizing activity, 1719 and 2278, were present by scRNAseq in memory B cells (MBC) or plasmablasts (PB) and when mAbs were isolated. **D.** 14 mAbs from lineage 1719 (including 9 previously characterized in [11]) and 5 mAbs from lineage 2778 were tested for neutralization against the Z1800M T/F Env PV and two mutants in which previously determined neutralization escape mutations were introduced into the gp120 V5 domain; D10.V5 in **E** and D11.V5 in **F**. Serially diluted mAb concentration is shown on a log10 scale plotted against percent viral infectivity. All 1719 mAbs neutralized the Z1800M T/F Env PV, including week 26 (n = 3, green), week 63 (n = 9, red), and week 90 (n = 2, purple). Note that the UW4-1 mAb is identical to the individualized germline allele, indicating that it is representative of the rearranged unmutated precursor for the lineage. The week 26 mAbs from lineage 2778 were poorly neutralizing (n = 2, green), while the week 55 (n = 1, asparagus) and week 63 (n = 2, red) mAbs neutralized much more potently. The human neutralizing mAb 1A8 isolated from Z1800M and the HIV-1 bnAb VRC01 were used as positive controls (blue); anti-influenza HA mAb EM4C04 and RLk17 non-neutralizing mAb 2B12 were used as negative controls (black). VRC01 neutralization is not affected by the V5 mutations, while the Z800M mAb 1A8 cannot neutralize the V5 mutants.

In our prior work, we administered SIVmac239 Gag encoding DNA at weeks 0 and 8 and MVA at weeks 16 and 24, both of which also expressed an HIV-1 T/F Env, either the clade A/C recombinant R66M Env or the clade C Z1800M Env (Fig 1) [11]. The Z1800M T/F Env was associated with robust autologous and heterologous responses during infection while the R66M T/F Env did not elicit either type of neutralizing activity [11,13]. These immunizations were followed by boosts at weeks 53 and 61 with the same Env presented as either monomeric gp120 or stabilized trimeric gp140 protein (Fig 1). We collected MBC and PB at peak time points during immunization to support an in-depth analysis of the B cell repertoire and dynamics (Fig 1), as well as to track the development of neutralizing antibodies. All animals developed high levels of serum antigen-specific IgG levels; however, only two animals, both having received the clade C Z1800M Env based DNA/MVA/gp120, developed autologous neutralizing activity against the autologous T/F Env PV (Fig 2A). Serum autologous neutralization activity was assessed using samples collected at weeks 18 (2 weeks post MVA1), 26 (2 weeks post MVA2), 55 (2 weeks post protein1), 63 (2 weeks post protein2), and 74 (13 weeks post protein2) (Figs 1 and 2A). Neutralization was first detectable in serum two weeks after the first protein boost for both RM; however, ROa17 developed only moderate activity that did not increase with a subsequent protein boost. Serum neutralization kinetics for RLk17 is shown with more detail in Fig 2B. Neutralization was not detectable using a 1:20 dilution of serum collected at weeks -4, 18, 26, or 53. Potent neutralization appeared shortly thereafter at week 55, increased after the second protein boost, and then waned but remained detectable for another 29 weeks without further immunization. RLk17 was therefore of significant interest due to a robust and durable neutralizing antibody response that appeared in serum after boosting with monomeric gp120 protein. Moreover, in both neutralization positive RMs, the vaccine elicited neutralizing antibodies targeted the same epitope as in the early clade C HIV-1 infection. A lack of glycan coverage in the gp120 V5 domain, proximal to the CD4 binding site (CD4bs), was associated with neutralization in infection and vaccination, and resistance to neutralization by antibodies induced in both settings was conferred by naturally occurring reorientation of an N-linked glycan in V5 [11]. These results demonstrated remarkable convergence between responses elicited in immunized RM and those of the person living with clade C HIV-1 from whom the T/F Env was isolated.

**Fig 3:**
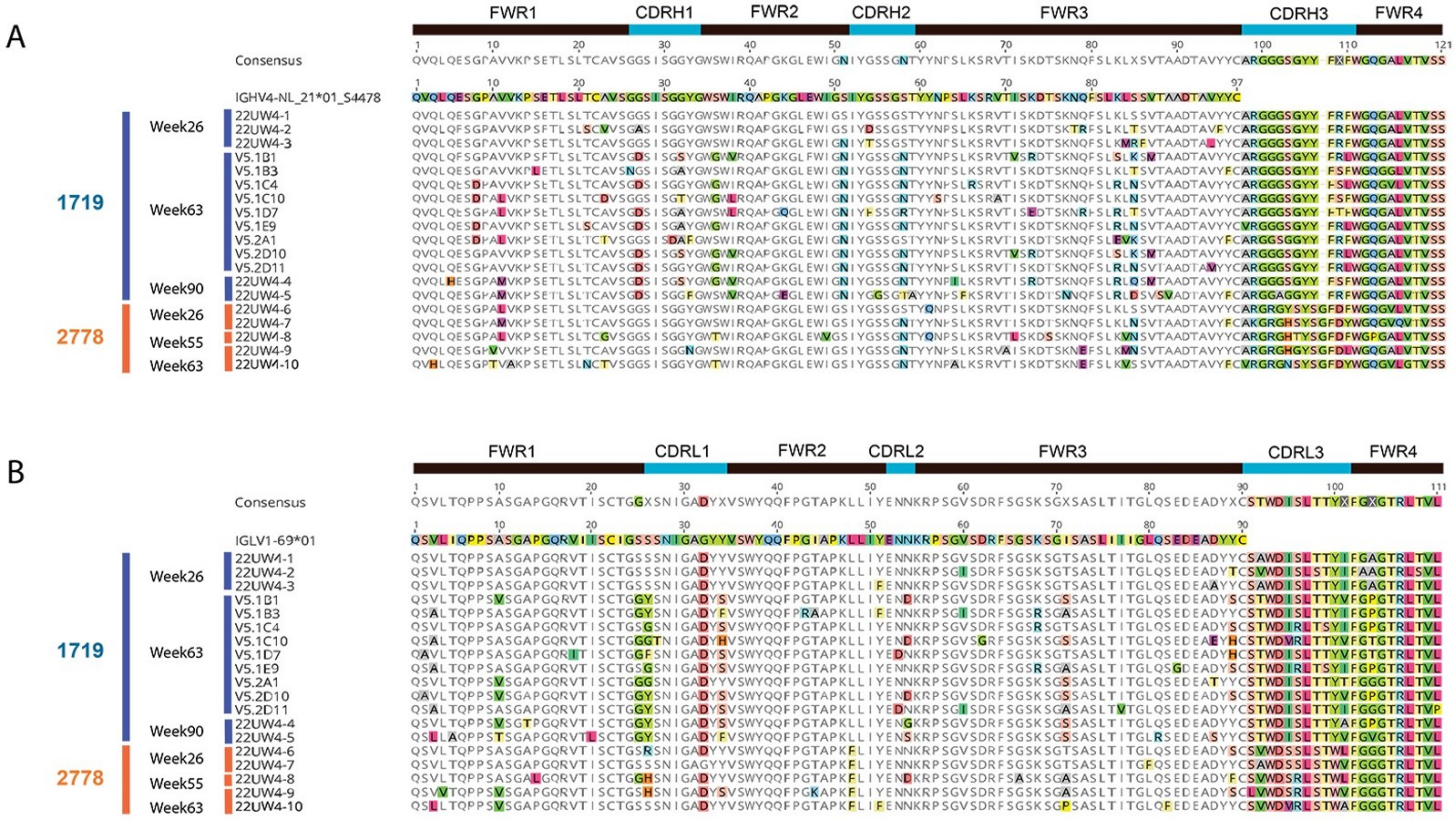
Amino acid alignments of the VH and VL regions for RLk17 1719 and 2778 lineage mAbs that were evaluated for neutralization. Amino acid sequences for 14 mAbs from lineage 1719, weeks 26, 63, and 90, and 5 mAbs from lineage 2778, weeks 26, 55, 63, are shown. The VH chains are shown in **A** and VL chains are shown in **B**. The germline allele is shown and used as the reference sequence, with amino acid differences highlighted in color. A consensus for all mAb sequences is shown above each alignment and the CDR1-3 regions are indicated.

We previously isolated nine neutralizing monoclonal antibodies (mAbs) from RLk17 by sorting antigen specific MBC that were cryopreserved at week 63 using a combination of wildtype and V5 mutated Z1800M T/F Env gp120 probes to enrich for neutralizing B cells [11]. Here, through single cell B cell receptor sequencing, we found that this clonotype (1719), was also present in MBC at weeks 26 and 90 and in PB at week 61 (Fig 2C). A second clonotype (2778) was also enriched by the week 63 wildtype/V5 mutant probe B cell sort, and it shared the same VH and VL germline alleles as 1719 (Fig 3A and 3B). This clonotype was also present in MBC at weeks 26 and 55 and in PB at week 61 (Fig 2C). The VH and VL of both lineages also shared features with a human neutralizing mAb, 1A8, isolated from Z1800M during early HIV-1 infection, such as their germline sequence, CDRH3 length, and level of SHM [11,15]. To explore the neutralizing trajectory of lineages 1719 and 2778, we generated novel mAbs from representative VDJ/VJ sequences present in MBC at weeks 26 and 90 for 1719 and weeks 26, 55, and 63 for 2778 (Fig 2C). The week 26 mAbs from lineage 2778 and 1719 exhibited a range of neutralizing activity against the autologous Z1800M T/F Env PV with IC_50_ titers ranging from 0.05 μg/ml to more than 25 μg/ml (Fig 2D). The neutralization potency of the antibodies increased over time, with mAbs reaching potency as high as an IC_50_ of 0.05 μg/ml at weeks 63 and 90 (Fig 2D). We also confirmed that all mAbs derived from the 1719 and 2778 clonotypes recognized the previously characterized V5 dependent/CD4bs proximal epitope [11]. All 1719 and 2778 mAbs from weeks 26, 55, 63, and 90 were unable to neutralize the autologous Z1800M T/F Env PV when resistance associated changes from the natural escape Env variants D10 and D11 were introduced into V5 (Fig 2E and 2F). Thus, these two lineages recognize the same or a highly similar epitope proximal to V5 and the CD4bs. Through high throughput sequencing, we found that the neutralizing lineage B cells persisted through multiple cycles of recall, SHM, and expansion through which they gained increasing neutralization potency.

### Defining the germline and evolutionary characteristics of the neutralizing B cell lineages

To better understand the parallel evolution and divergence of the neutralizing lineages, an individualized immunoglobulin germline database was constructed for RM RLk17 using IgDiscover [16]. However, only V gene alleles were produced by IgDiscover for the individualized database. We therefore combined the individualized database and a database from Cirelli *et al*. [3], which contains V, D, and J alleles, to annotate the VH chains of the RLk17 neutralizing lineages. This analysis supported that the IGHV4_NL_21*01_S4478 germline allele gave rise to both the 1719 and 2778 lineages with the highest V gene identities of 100% and 98.99%, respectively. This allele was also described previously and is present in the Karolinska Institutet Macaque Database (KIMDB) [17]. Individualized databases for the other 19 RM were also constructed using IgDiscover with the purpose of determining whether the IGHV4_NL_21*01_S4478 allele was present (see S1–S20 Files). This allele was present in three other immunized RM besides RLk17; however, none of those animals received the Z1800M Env containing vaccines (S1 Fig). Given the high divergence between the Z1800M and R66M T/F Envs, including major differences in the arrangement of putative N-linked glycans, it is unlikely that the R66M T/F Env displayed the same glycan hole around V5/CD4bs as the Z1800M Env [11,13]. The inability of RLk17 serum to cross-neutralize the R66M T/F Env PV supports this assumption [11].

**Fig 4:**
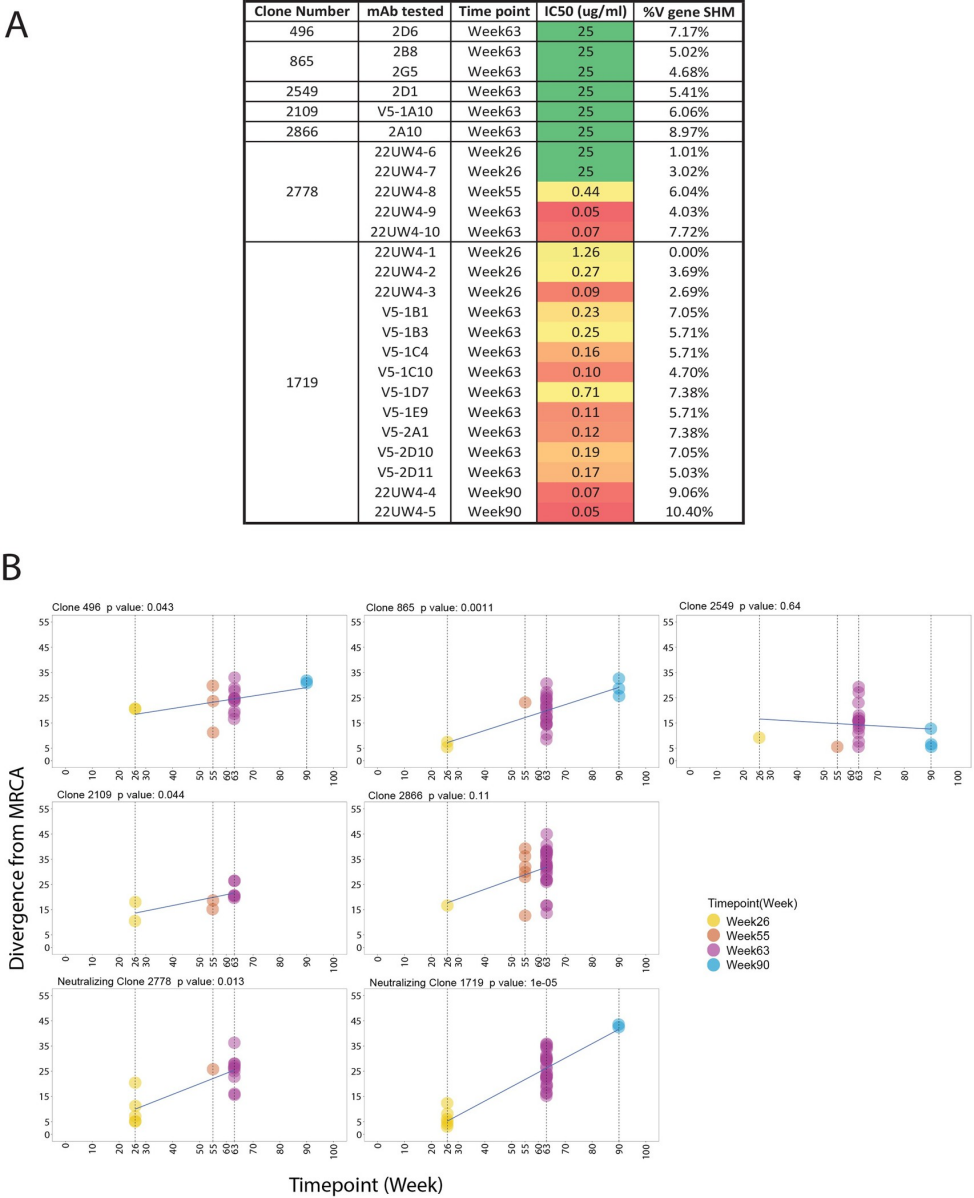
Divergence from the most recent common ancestor by neutralizing and non-neutralizing B cell lineages in RLk17. **A.** The percent of SHM in the V region of the VH chain compared to the individualized germline allele is shown alongside the IC_50_ neutralization titer against the Z1800M T/F Env PV for 6 mAbs that bound to gp120 in ELISA but were non-neutralizing (2D6, 2B8, 2G5, 2D1, V5-1A10, 2A10), ranging from 4.68% to 8.97%. These mAbs were isolated at week 63 through antigen-specific B cell sorting representing 5 different B cell clonotypes. The same information is shown for lineage 2778 and 1719 mAbs isolated at weeks 26, 55, 63, and 90. At week 63, the percent of SHM for 2778 was 4.03% to 7.72% and for 1719 was 4.70% to 7.38%. The color gradient from green to red indicates ID_50_ titers from undetectable to low to high potency. **B.** Divergence from the most common recent ancestor (MRCA) for the 5 non-neutralizing and 2 neutralizing clonotypes in panel A is plotted over time. Each graph represents a B cell clonotype and the p value indicates whether the trajectory of divergence was significant across time points, with p < 0.05 considered significant using a date randomization test. Each point represents a VH chain V region sequence present in MBC for that clonotype. Non-neutralizing clonotypes 496, 865, 2109 and neutralizing clonotypes 1719 and 2778 showed significant divergence from the MRCA over time. Time points started at week 26 for all clonotypes and extended through at least week 63.

We next examined the sequences of the mAbs tested for neutralization by determining their identity to the individualized VH germline for the 1719/2778 lineages. Compared to the inferred VH germline, the week 26 mAb 22UW4-1 had 100% identity at the amino acid and DNA levels (Figs 3A and 4A). Unexpectedly, this mAb neutralized the autologous Env PV with an IC_50_ of 1.3 μg/ml, which was roughly two-fold less potent than the 1719 mAb V5-1D7 obtained at week 63 with an IC_50_ of 0.71 μg/ml and estimated V gene SHM of 7.4%. Likewise, the VL chain of mAb UW22UW4-1 had only one amino acid difference from the assigned VL1-69*01 germline, and this glycine to aspartic acid substitution was present in all other 1719 mAb sequences (Fig 3B). Week 26 neutralizing mAbs 22UW4-2 and 22UW4-3 neutralized the autologous Env PV with IC_50_ titers of 0.27 and 0.09 μg/ml with little divergence from germline. Notably, the three 1719 week 26 mAbs had identical third complementarity determining regions in their heavy chains (CDRH3) (Fig 3A), demonstrating that the substantial differences in neutralization potency were driven by mutations outside of that region. The week 26 mAb 22UW4-2 had only 8 amino acid differences from germline located in the first framework (FRW1) (n = 2), CDRH1 (n = 1), CDRH2 (n = 1), and FRW3 (n = 4) of the V region with 3.7% SHM from the germline DNA sequence (Figs 3A and 4A). The 1719 week 26 mAb 22UW4-3 was even more potent but paradoxically had fewer amino acid differences from germline (n = 6) in CDRH2 (n = 2) and FRW3 (n = 4) and was less mutated at 2.7% V gene SHM (Figs 3A and 4A). The VL chains for mAbs 22UW4-2 and 22UW4-3 had two and three amino acid differences from germline, respectively. The low divergence from germline in both the VH and VL chains of the three 1719 week 26 mAbs supports their recent emergence. Furthermore, the finding that a putative rearranged unmutated precursor of lineage 1719 was capable of autologous neutralization is remarkable. The presence of these neutralizing mAbs with high identity to germline at week 26, two weeks after the second MVA immunization, argues that the 1719 lineage was activated by this vaccine component at either week 16 or 24. In the other neutralizing lineage, 2778, the week 26 mAb 22UW4-6 had only two amino acid changes from germline in FRW1 (n = 1) and FRW3 (n = 1) with only 1% SHM in the V gene (Figs 3A and 4A). Likewise, week 26 mAb 22UW4-7 had four amino acid differences from germline in FRW1 (n = 1), CDRH2 (n = 1), FRW3 (n = 2) and had 3% SHM (Figs 3A and 4A). In contrast to the 1719 week 26 mAbs, the corresponding 2778 mAbs exhibited weak neutralization of the autologous Env PV, with IC_50_ titers above 25 μg/ml. The VL chains for the 2778 week 26 mAbs also differed from germline by only 2 or 3 amino acids (Fig 3B), supporting their recent emergence.

**Fig 5:**
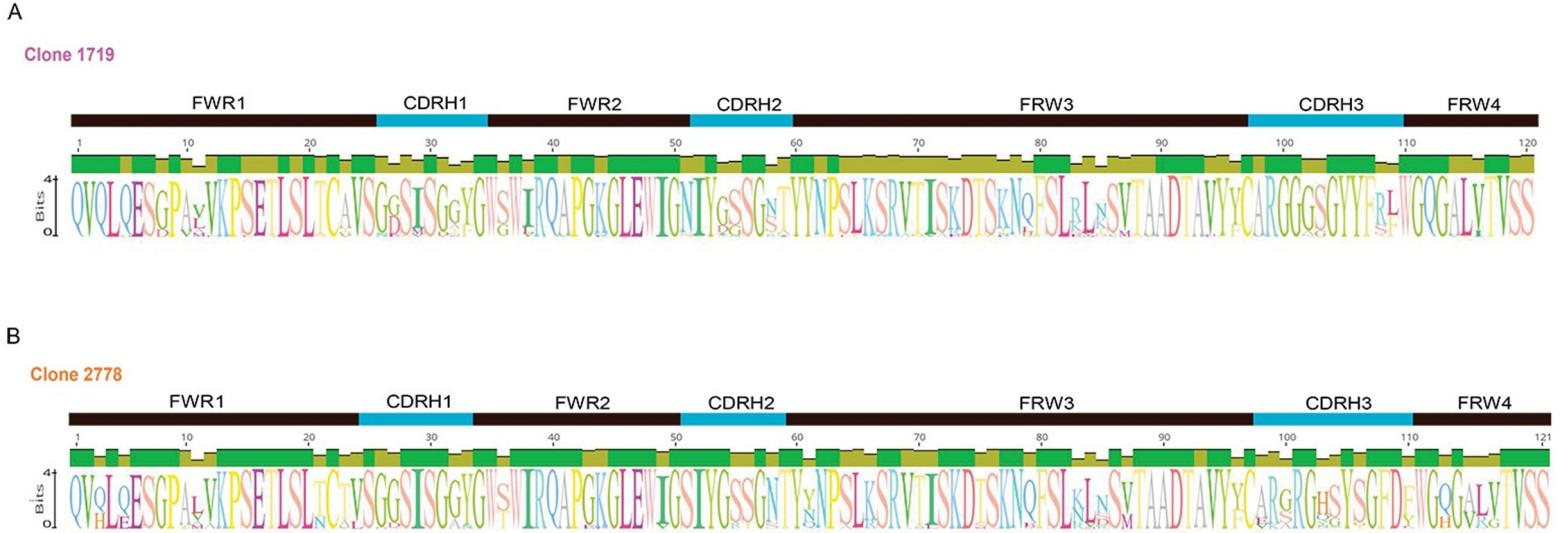
Sequence logos summarize amino acid changes in the RLk17 neutralizing lineages 1719 and 2778 over time. A sequence logo was generated for each lineage in Geneious Prime v2023.2.1 using an alignment of all available sequences at weeks 26, 55, 63, and 90. An identity graph, representing sequence conservation at each position, is also shown above each logo. Lineage 1719 is shown in **A** and 2778 is shown in **B**. Amino acids occurring at each position are indicated by the single letter codes, with height representing frequency. The regions of the Ig variable domain are annotated above the logos.

The mAbs of both lineages tended to increase in neutralization potency at the later time points (Figs 2D and 4A), coinciding with divergence from germline through amino acid sequence entropy across both the FRW and CDR regions (Fig 3). The most potent lineage 2778 neutralizers were the week 63 neutralizing mAbs 22UW4-9 and 22UW4-10, with IC_50_ titers of 0.05 and 0.07 μg/ml that corresponded to 4.0 and 7.7% SHM in the V gene, respectively (Figs 3A and 4A). 22UW4-9 had only seven amino acid changes from germline in the VH chain while 22UW4-10 had 11 differences. 22UW4-9 and 22UW4-10 shared only two amino acid substitutions in common, indicating that multiple pathways led to greater neutralization potency. The most potent of the 1719 lineage mAbs was 22UW4-5, which was isolated at week 90 with an IC_50_ titer of 0.05 μg/ml and 10.4% SHM in the V gene (Figs 3A and 4A). This mAb was the most divergent variant from either lineage, with 16 amino acid substitutions in VH. Several 1719 mAbs from weeks 26 and 63 had IC_50_ titers around 0.1 μg/ml with SHM ranging from 2.7% to 7.4% in the V gene (Figs 3A and 4A). Thus, multiple lines of evidence suggest that acquisition of neutralization potency was not linear and appears to have involved multiple evolutionary trajectories, varying levels of SHM, and exploration of the paratope space within and across clonotypes. Examining all available sequences from each lineage demonstrates that distinct patterns of SHM occurred (Fig 5). Fig 5A demonstrates that positions within FWR1, CDRH1, FWR2, CDRH2, FWR3 were under strong selection in 1719 and multiple positions in these regions became fixed over time, while others did not. Interestingly, the CDRH3 sequence exhibited toggling between amino acid residues at positions 108 and 109, and some week 63 variants had a CDRH3 that was the same or very similar to that present at week 26. In 2778, positions in FRW1, CDRH1, CDRH2, FRW3 and FRW4 were also under strong selection, with CDRH3 again toggling at multiple positions (100, 103, 104 and 110), and showing similarity to early variants at later time points (Fig 5B). These observations illustrate that amino acid substitutions over time within these neutralizing antibody lineages did not always become fixed but rather exhibited continued sampling. Furthermore, co-selection directed at the FRW and CDR regions was observed in concert with increasing neutralization potency, consistent with affinity maturation and the need to balance the direct antigen contact of the CDRs with the stabilizing effects of FWR regions.

### Both neutralizing and non-neutralizing clonal lineages evolve by acquiring SHM during immunization

To quantify the longitudinal evolution of the neutralizing 1719 and 2778 lineages, a modified phylogenetic date randomization test was utilized [18,19]. We compared the 1719 and 2778 clonotypes to five others in the same animal, RLk17, that produced mAbs that bound to Z1800M T/F Env gp120 in ELISA but lacked autologous neutralizing activity (496, 865, 2549, 2109, 2866) (Fig 4A). All non-neutralizing clonotypes were present at weeks 26 and 63 with at least one additional time point (Fig 4B). The two neutralizing clonotypes, 1719 and 2778, exhibited significant divergence over time (p = 1e-05 and p = 0.013, respectively) (Fig 4B). However, non-neutralizing clonotypes 496, 865, and 2109 also underwent significant levels of divergence from the inferred most common recent ancestor (MRCA) (p = 0.043, 0.0011, and 0.044, respectively). Finally, the two other non-neutralizing clonotypes, 2549 and 2866, did not evolve significantly from weeks 26 to 63 or weeks 26 to 90 (p > 0.05) (Fig 4B). Thus, SHM in the non-neutralizing B cell lineages could potentially increase affinity for epitopes that are not found on the native Env trimer but are presented by the immunogens trapped within germinal centers. This finding highlights the difficulties in eliciting neutralizing antibodies within a sea of non-neutralizing B cell specificities that are also likely to be undergoing rounds of SHM.

**Fig 6:**
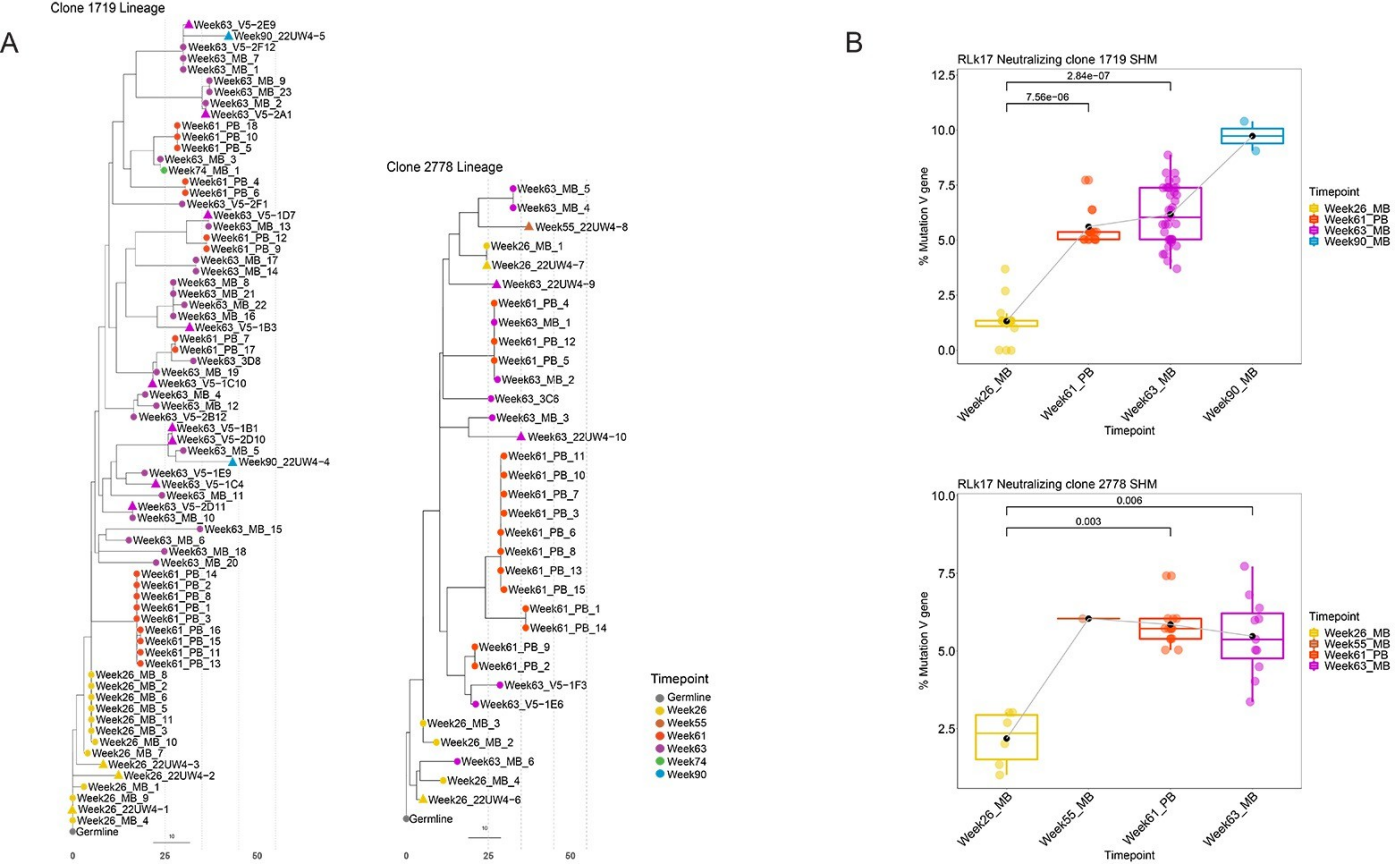
Diversification of neutralizing lineages 1719 and 2778 over time in RLk17. **A.** Phylogenetic trees were constructed for all available VH chain sequences from each clonotype, MBC sequences from weeks 26, 55, 63, 74, 90, and PB sequences from week 61, coded by color. Triangles indicate that mAbs with the corresponding sequence have been tested and they neutralized the Z1800M T/F Env PV. The grey dotted lines indicate 10 estimated nucleotide mutations from the most recent common ancestor. **B.** The percent mutation in the VH chain V gene compared to the individualized germline is shown for the same MBC and PB sequences as in the trees in panel A. Significantly higher SHM was observed at week 61 and 63 compared to week 26 for both lineages using a Wilcoxon rank sum test. A p value < 0.05 was considered significant.

To visualize divergence from germline at higher resolution, maximum likelihood trees were generated for all sequences from the neutralizing lineages using Dowser [19] and the pml method. Phylogenetic trees are shown in Fig 6A, with each node corresponding to a unique B cell receptor sequence within the 1719 and 2778 lineages. The branch lengths represent the SHM per site in the V region of the VH chain sequences and their sum represents divergence from the MRCA (Fig 6A). These trees demonstrate increasing divergence over time in both neutralizing lineages, with the week 90 variants of 1719 acquiring the greatest distance from the MRCA (Fig 6A). We next statistically analyzed changes in SHM over time in the two lineages. Comparisons between time points demonstrated highly significant increases in SHM in antigen specific MBC and PB compared to week 26 for both neutralizing lineages (p < 0.05 by Wilcoxon test) (Fig 6B). In 1719, the parallel gain in neutralization potency compared to 22UW4-1 was 5 to 10-fold in the week 26 variants, 2 to 10-fold in week 63 variants, and 10 to 18-fold in week 90 variants that were tested as mAbs (Figs 2D and 4A). In 2278, week 55 and week 63 variants gained 2–3 logs of neutralization potency compared to the poorly neutralizing week 26 variants (Figs 2D and 4A).

### Clonal representation and overlap

**Fig 7:**
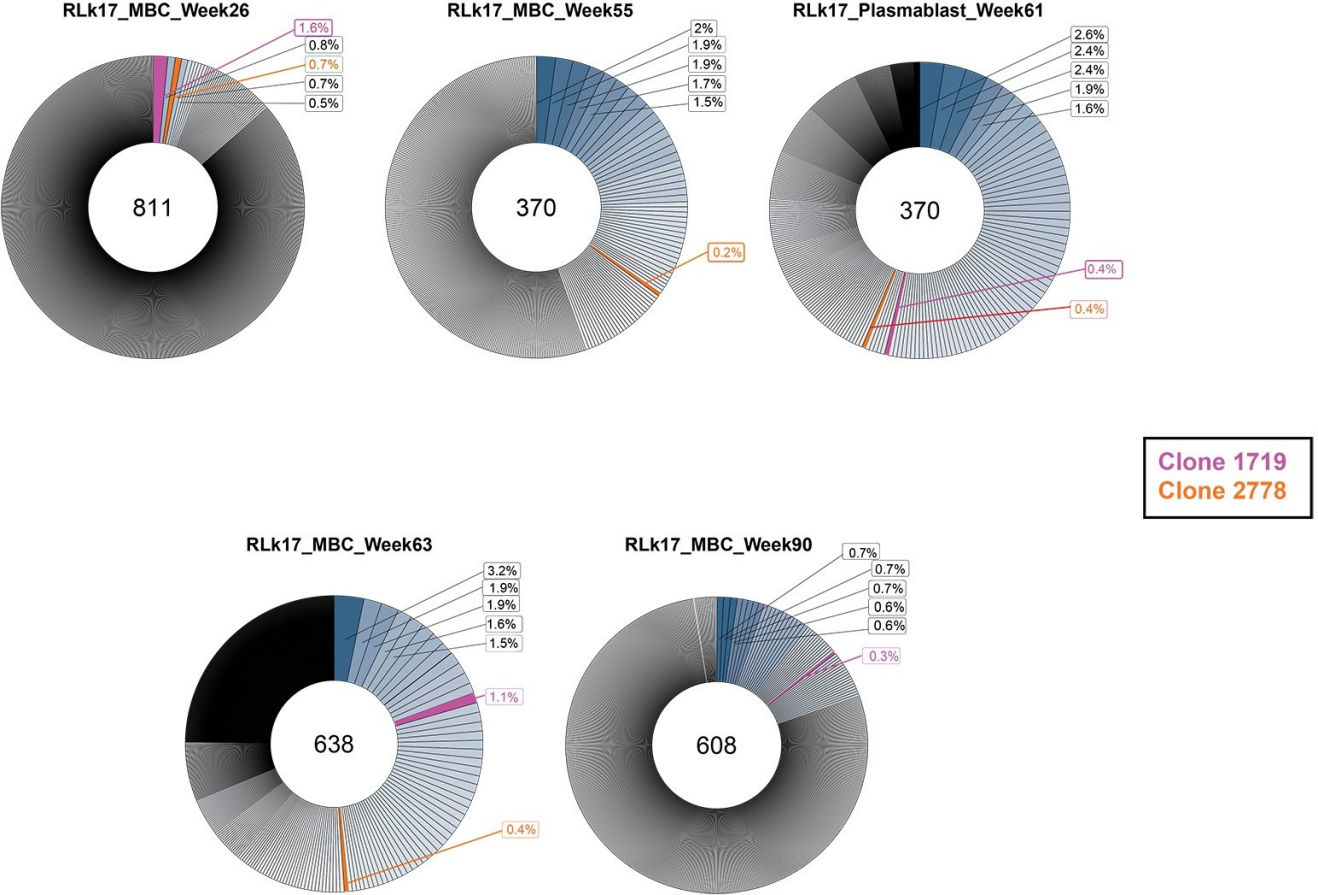
Clonal expansion in antigen specific B cells during immunizations. Donut charts representing the total number of B cell clonotypes (center) from RLk17 at each timepoint and the relative frequency of each individual clonotype in MBC at weeks 26, 55, 63, 90 and in PB at week 61. The top 5 most abundant clones are indicated, and frequencies of the neutralizing lineages 1719 and 2778 are shown in magenta and orange, respectively. 1719 was not detected at week 55 and 2778 was not detected at week 90.

To track the dynamics of the 1719 and 2278 neutralizing lineages over time in the neutralizing RM RLk17, we analyzed sequences from high throughput RNA VDJ/VJ repertoire sequencing on acute CD80+ PB and circulating CD20+ IgG+ antigen specific MBC (Fig 7). PB were analyzed from blood collected at four days after DNA2 (week 8), MVA2 (week 24), protein1 (week 53), and protein2 (week 61) immunizations and VH sequences were analyzed by using immunoglobulin repertoire sequencing (RepSeq). For MBC, antigen specific B cells were sorted from cryopreserved PBMC collected two weeks after the DNA2 (week 10), MVA2 (week 26), protein1 (week 55), protein2 (week 63), and necropsy (week 90). A summary of the high throughput sequencing results for RLk17 MBC and PB is shown in Fig 7. At two weeks after MVA2, clonotypes 1719 and 2778 were among the top three expanded clonotypes in RLk17. The frequency of 1719 was 1.6% of all clonotypes, with 2778 comprising 0.7%. Thus, of 811 individual antigen specific B cell clones at this time point, 13 belonged to the 1719 lineage and 6 belonged to the 2778 lineage. At week 55, two weeks after protein1, no 1719 members were detected but 2778 was present at a frequency of 0.2%. Four days after the protein2 at week 61, both families were present at frequencies of 0.4% in the PB compartment. Note that we also examined PB at four days after the DNA2, MVA2, and protein1, but did not detect either of these lineages circulating in that compartment. Interestingly, week 61 coincided with the strongest observed serum neutralization activity for RLk17 appearing two weeks later (Fig 2B). At that time (week 63), both lineages were also present at relatively high frequencies in MBC: 1.1% for 1719 and 0.4% for 2278. Finally, 29 weeks after the final immunization, we detected only lineage 1719 at week 90 at a frequency of 0.3%. Clonotype 1719 appeared to be more persistent, potentially due to gaining higher affinity binding to antigen in the germinal centers, whereas 2778 may have been competed out. Thus, lineages competing for the same target region or epitope could be a mechanism that drives eventual selection for higher affinity clonotypes.

**Fig 8:**
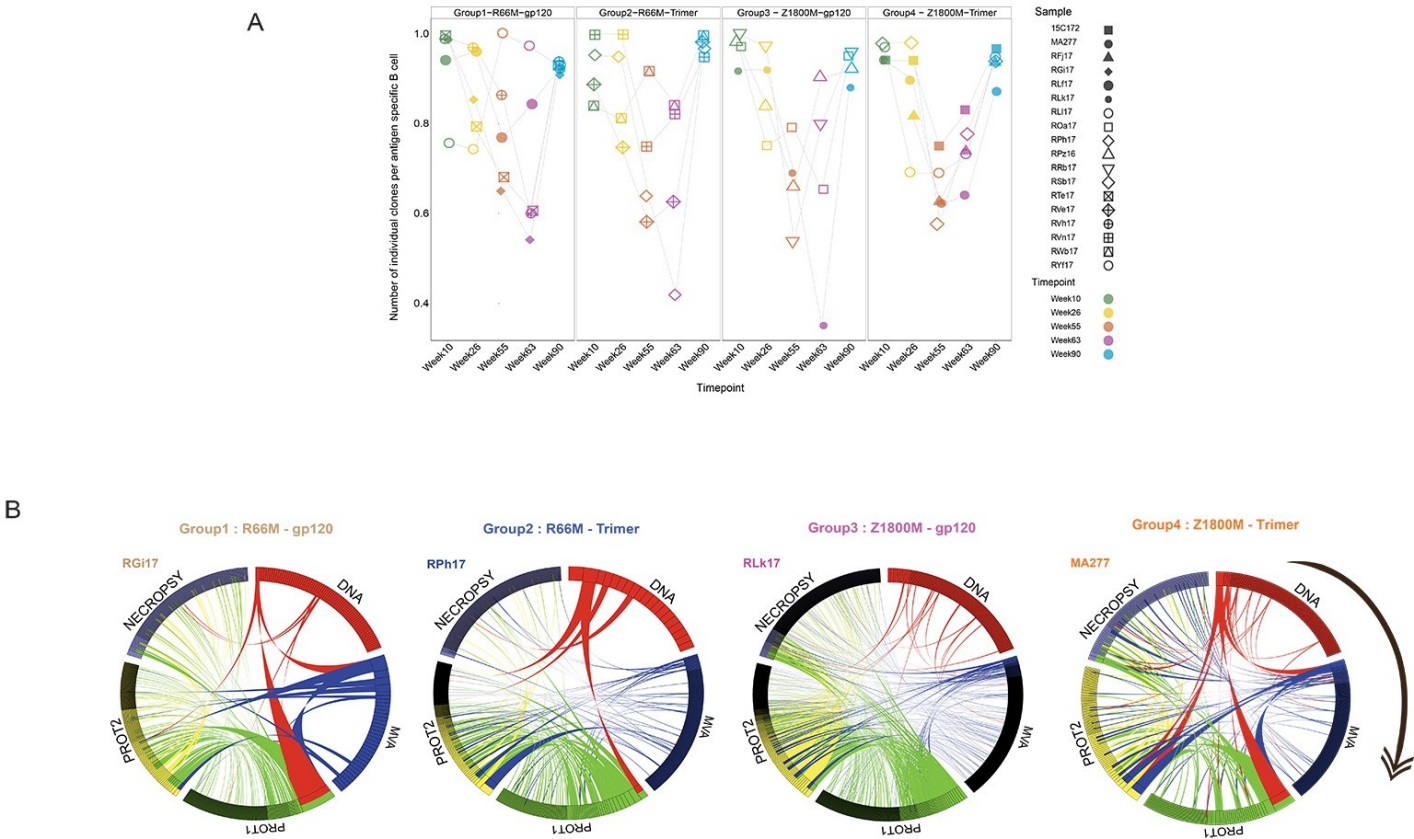
Clonal representation and overlap of antigen specific B cells during immunizations. **A.** The number of individual clonotypes per cell sequenced is plotted for animals in each vaccination group in MBC at weeks 10 (second DNA), 26 (second MVA), 55 (first protein), 63 (second protein), and 90 (necropsy), organized by vaccination group. Values closer to 1 indicate more singlets while lower proportions indicate more clonotypes that had multiple clonal members. **B.** Circos plots illustrate the overlap and recall of antigen specific MBC throughout immunization. A representative animal for each group is shown, with all animals shown in S2 Fig. Ribbons are color coded as follows: second DNA = red, second MVA = blue, first protein = green, second protein = yellow and this order represents the sequence of vaccination and necropsy. The color of each ribbon indicates the immunization time point at which the clonotype was first identified. RLk17 is the only animal that developed persistent neutralizing antibodies.

The ability of responding antigen specific B cells to expand could vary by Env immunogen or could be important for developing neutralizing antibodies. We therefore examined the overall number of distinct B cell clonotypes in isolated Env-specific MBC across all vaccinated animals (Fig 8A). The number of clonotypes was normalized across animals and time points by dividing this parameter by the number of B cells with paired VH and VL sequenced for each sample. This proportion would approximate whether responding B cells consisted mainly of singlets or of clonotypes that had expanded to multiple clonal members. Overall, responding B cells followed multiple trends in individual animals but expansion was generally observed after the MVA and protein immunizations. Notably, antigen specific MBC in RLk17 (small, filled circles, group 3) at week 63 contained the highest proportion of expanded clonotypes across all animals, although the top five most abundant clonotypes did not include the neutralizing lineages (Fig 7). However, RPh17 in group 2 (open diamonds) followed a similar trend as RLk17 yet did not develop serum neutralizing activity. An interesting observation is that antigen specific B cells in all group 4 animals, which were immunized with the Z1800M Env trimer, failed to expand after the second protein boost. However, there was not a discernible impact of the different Env immunogens.

We next explored how antigen specific B cell clonotypes persisted and overlapped across time points. In Fig 8B, a Circos plot depicting clonotype recall is shown for a representative RM from each vaccination group, with RLk17 being the only one that developed serum neutralizing activity. Plots for all RM are shown in S2 Fig. Antigen specific B cell clonotypes that were present following DNA2 vaccination were boosted at a relatively low level by subsequent immunizations; however, some persisted in the MBC compartment until necropsy, 80 weeks after DNA2. Extensive overlap of clonotypes between the protein1 and protein2 immunization time points was observed; however, we did not perform this analysis between DNA1-DNA2 and MVA1-MVA2 time points. Despite this caveat, there does seem to be a trend of greater clonotype persistence after the protein immunizations, and the lack of connection between DNA2, MVA2 and the later timepoints is notable. The level or pattern of clonotype recall did not appear to differ across vaccination groups or reflect whether neutralizing antibodies developed. However, the majority of these clonotypes are likely to represent non-neutralizing specificities that could interfere with the maturation of neutralizing B cell lineages if present.

## Discussion

Current HIV vaccines in preclinical and clinical pipelines do not reliably elicit strain specific or broadly neutralizing antibody tier 2 responses in nonhuman primates or people using standard administration routes. Indeed, only a handful of HIV-1 Env based vaccines have achieved this milestone in nonhuman primates or human volunteers, and this small group includes our Z1800M Env-based vaccine [11,12,20–22]. Neutralization responses elicited in humans against the most advanced Env trimer immunogen BG505 SOSIP have been modest at best, highlighting that there are fundamental questions still to be answered [23]. It is therefore critical to understand mechanisms by which neutralizing antibodies arise, persist, and mature in response to HIV-1 vaccination in relevant models such as RM. We successfully elicited autologous tier 2 neutralizing antibodies against a clade C T/F Env in the absence of a stabilized Env gp140 trimer and focused on one RM that developed durable high titer neutralizing antibodies to learn more about how this response evolved. Two independent neutralizing lineages arose from the same VH and VL, and for one of the lineages we isolated a clonal member that is representative of the unmutated rearranged B cell precursor, which had autologous neutralizing activity. The earliest detection of both lineages in MBC was week 26, which was two weeks after the second MVA immunization. As germinal centers typically peak around 2 weeks post-immunization, it seems likely that these lineages were engaged by the first MVA immunization. Furthermore, neither lineage was detected in MBC or PB after the second DNA immunization, although there were fewer antigen specific B cells and low serum IgG responses at this time point overall, consistent with previous studies [8,24,25]. The protein immunizations resulted in higher SHM and ID_50_ titers for both lineages but also higher SHM for non-neutralizing B cell clonotypes. Eliciting a durable, strain specific or broad neutralizing antibody response is not just an issue of inducing SHM; indeed, we and others demonstrate that priming and boosting with HIV-1 Env vaccines drives expansion, affinity maturation, and broad anatomical dissemination of a large number of antigen specific B cells, most of which are not neutralizing [26]. A successful vaccine will require optimization of immunogen and epitope, and early engagement of capable B cells with a sufficient precursor frequency from among a diverse immunoglobulin repertoire. Engaged lineages will then need to progress through random and poorly understood SHM pathways, while competing with a sea of non-neutralizing B cell counterparts. Understanding these processes is fundamental to progress towards a broadly protective vaccine. Strategies involving prolonged Env trimer delivery have demonstrated benefits such as antigen specific MBC trafficking through multiple germinal centers, acquiring higher autologous neutralization titers and possibly a hint of serum heterologous neutralizing capacity [2]. Thus, translating these advances will be important. In our study, the data suggests that both neutralizing and non-neutralizing clonotypes continued to persist and mutate over a long period of time although we did not use a slow antigen delivery method. Other studies of Env specific B cell lineages in RM reported the presence of many lineages in peripheral blood, draining lymph node, and spleen, consistent with ongoing germinal center responses following boosting [20,26]. That two neutralizing lineages were likely initiated by MVA immunization, despite no detectable serum neutralization and prior to protein boosts, suggests that a better understanding of this type of platform is needed to capitalize on its advantageous features. Our previous study revealed that the DNA/MVA/gp140 plus 3M-052 adjuvant vaccine regimen produced the best tier 2 serum neutralization activity across 7 different preclinical trials that all expressed the clade C Env 1086.C in RM. Furthermore, Eslamizar *et al*. demonstrated that MVA prime followed by a clade C Env gp120 boost induced higher frequencies of germinal center B cells expressing peanut agglutinin (PNA), as well as antigen specific B cell and T follicular helper responses, compared to DNA and VSV primes [27].

The recombinant DNA and MVA modalities that we employed have been used to deliver multiple immunogens for pathogens including HIV-1, SIV, TB, and SARS-CoV-2 [11,28–34]. The MVA constructs co-express full-length Gag Pr55 with Env gp145, which promotes formation of viral-like particles (VLPs) that contain Gag and display HIV-1 Env on the surface [24]. MVA encoded HIV-1 Env gp145 has been detected on the surface of VLPs, MVA-infected cells, and the MVA virion *in vitro* [35]. These various membrane-bound presentations of Env potentially present a form of the trimer *in vivo* that is closer to that seen on virions. While MVA does not replicate in human cells [36], it does produce extracellular vesicles [37] and replicates in some nonhuman primate cell lines [38], raising the possibility of persistent antigen. A more comprehensive characterization of the recombinant MVA virus particle, Gag-Env VLPs, and Env expression on infected cells using our vaccine is warranted, as is the development of updated delivery strategies to present native Env and extend germinal center duration, such as novel nucleic acid-based approaches. If MVA presented a native trimer-like Env form, it was somewhat surprising that monomeric gp120 was effective at boosting neutralizing antibodies. While the gp120 monomer exposes non-neutralizing surfaces on the inner domain, it also expresses epitopes shared with the virion associated trimer and lacks the immunodominant base present in stabilized gp140 trimers that overwhelmingly elicits non-neutralizing antibodies. Indeed, in our experience, Env gp120 probes more efficiently bait neutralizing B cells than trimer probes [11,12]. Interestingly, human immunizations with monomeric gp120 alone or combined with heterologous vectors have failed to elicit tier 2 neutralizing antibodies in clinical trials [39–43]. Our results suggest that both the Env immunogen and the priming/boosting agents are key determinants of the antibody response and should be considered carefully. The first immunization in a prime-boost vaccination is likely to be critical for how the immune response unfolds [44].

A longstanding dogma for human antibodies is that mutations in the FRW are poorly tolerated and when they do occur are skewed towards neutral substitutions that maintain structural integrity [45,46]. This is related to a higher level of degeneracy in codons used in the FRWs; in contrast, mutations that significantly alter the amino acid sequence occur preferentially in CDRs [47]. Thus, FRW substitutions are constrained because B cells that fail to express a functional BCR will not be selected for survival [48]. Human antibodies in general will accumulate 10–15 somatic mutations that occur mainly in the CDR regions [49]. We were able to track two vaccine induced neutralizing antibody lineages with high identity to the individualized germline in one RM, which mimicked human neutralizing antibody responses against HIV-1 Env. Examination of SHM and increasing neutralization potency over an extended period demonstrated that mutations readily accumulated in both FRW and CDR regions. For antibodies against HIV-1 Env, changes in the FRW regions have been shown to contribute to neutralization breadth and potency in the context of bnAbs [50–53]. FRW changes in this context have been shown to involve direct engagement with antigen or exert an indirect impact on integrity, structure, and stabilization while the CDR regions evolve. One study demonstrated that FRW mutations in early variants of the HIV-1 bnAb CH103 lineage had a large impact on affinity [53]. FRW mutations have also been shown to increase antibody rigidity or flexibility during bnAb evolution, depending on the precursor affinity [51]. Klein *et al*. demonstrated that FRW mutations are essential for bnAb neutralization but did not contribute to weakly neutralizing antibody activity [54]. Our analysis followed antibody evolution longitudinally and linked accumulation of FRW and CDR changes with increases in neutralization potency in this vaccination setting, consistent with that reported in RM immunized with an Env trimer [26]. Our study contributes to the understanding of how vaccine-elicited neutralizing antibodies against HIV-1 Env initiate and mature over time [26,55,56]. The fixation of some changes in the FRW of lineages 1719 and 2778 suggests that they were under strong co-selection during affinity maturation, along with the CDR regions. The individual contributions of the FRW and CDR changes in 1719 and 2278 to binding affinity and neutralization potency warrant further investigation.

In addition to tracking the neutralizing B cell lineages, we are among the first to characterize antigen specific MBC and PB repertoire dynamics in HIV-1 Env immunized RM using two distinct Env immunogens. In terms of antigen specific properties that we examined; differences induced by the individual Env immunogens were not apparent. Expansion and persistence of many antigen specific clonotypes was observed in all RM regardless of Env immunogen or protein boost, with significant SHM over time, without producing detectable serum neutralizing antibody in most animals, consistent with a previous study using Env trimer immunization [26]. Some B cells could have had neutralizing potential or produced neutralizing antibody that went undetected; in fact, the former scenario likely occurred in ROa17 where we observed serum neutralizing activity that did not increase with subsequent protein boosting. Thus, a lack of detectable serum neutralization does not preclude the presence of neutralization capable B cells, as we isolated neutralizing B cells from week 26 in RLk17, a time when serum neutralization was not detectable in our assay. It may be that production of antibody secreting cells had not yet reached a threshold of detection at the time of sampling. We observed both persistent and unique antigen specific clonotypes arising at each immunization, but neutralization appears to have been mediated by B cells that arose early with MVA, which was the third and fourth immunization, and continued to mature throughout two subsequent protein boosts and beyond. It is curious that the V5/CD4bs target in our study was not masked and appeared to be available throughout for affinity maturation, although the 2778 lineage was not detected in MBC at week 90. For HIV-1, it may be important to engage neutralizing B cell lineages early before masking and competition can impede their selection and survival. We also did not observe any persistent VH germline bias in the immunized RMs, although the few neutralizing antibodies we isolated all arose from the same VH and VL germlines. Our data suggest that early engagement of neutralizing antibody precursors is likely to be a key element for successful competition against non-neutralizing B cells. Boosting recalls antigen specific B cells and this overwhelmingly drives non-neutralizing B cells to expand and mature. The factors that allowed these neutralizing lineages to gain traction in RLk17 could be many, including a high initial frequency of a suitable precursor with high binding affinity to antigen, and efficient and persistent germinal center formation, as germinal centers have been shown to correlate with autologous neutralization [3]. Our data also suggest that a scenario in which neutralizing B cells engage antigen but fail to sufficiently expand and persist differs from never developing any neutralizing B cell lineages, a distinction with significant implications.

We have also isolated autologous neutralizing mAbs from BG505 SOSIP immunized RMs using sorting of single antigen specific B cells [12]. Previously we described mAbs 1B1, 1B6, 1F3, and 1G3, isolated from one RM that developed potent serum neutralization targeted at a prominent glycan hole near V5 on gp120. All mAbs belonged to an expanded clonotype, arose from germline VH4.39C*01/VK1.15 pairing, and exhibited similar levels of potency, divergence from germline, but had a shorter CDRH3 region (9 vs 12–13 amino acids) than those reported in the current study. A potentially important difference is that B cells were presented with only the BG505 SOSIP immunogen as opposed to the varying forms of the Z1800M T/F Env. However, the BG505 neutralizing lineage was also expanded and persistent. Inspection of the VH amino acid sequences aligned with each germline demonstrated that SHM was focused mainly in the CDRH1, CDRH2, and FRW3 regions. In addition, each isolated mAb had a unique CDRH3 sequence. Thus, the mAbs had acquired potent autologous neutralizing capacity through moderate SHM in the CDR regions but also in FRW3. All these mAbs recognized the same target on the BG505 Env, the C3/465 glycan hole, but they exhibited varying sensitivity to addition, removal, and shifting of proximal glycans [12]. In contrast, all mAbs isolated from RLk17 were equally sensitive to the naturally occurring escape mutations that closed the glycan hole near the V5/CD4bs. These results are consistent with BG505 SOSIP being variably glycosylated proximal to the exposed region while the Z1800M T/F Env is more homogeneous in the V5/CD4bs neutralization target area. Furthermore, BG505 SOSIP has multiple, well characterized glycan holes that are targeted by neutralizing antibodies while Z1800M appears to have a single dominant glycan exposed region [12,57]. Taking the results of these studies and other nonhuman primate immunization studies together demonstrates that there are multiple germlines present in genetically diverse RM that can develop neutralizing activities against different HIV-1 Env immunogens [11,12,26]. These studies also demonstrate that neutralizing mAbs elicited by HIV-1 Env vaccination all exhibit standard size CDRH3 regions, moderate to high SHM in the V gene, and SHM in both FRW and CDR regions. In the case of Z1800M Env vaccination, the neutralizing mAbs we isolated are also like their human counterparts from infection.

Optimizing exposure and presentation of a known target region on an HIV-1 Env immunogen, such as the CD4bs proximal epitope exposed on the Z1800M T/F Env, could more efficiently engage precursors that can develop into expanded, persistent neutralizing antibody lineages. Indeed, the RLK17 vaccine induced mAbs tested to date competed with VRC01 for binding to the Z1800M T/F Env [11]. However, a thorough understanding of the immunogen, the immunoglobulin repertoire, and the antibodies that are elicited will be required. Our study supports that pre-focusing the B cell response on a desired epitope early, using delivery methods that present membrane bound trimers, could favor engaging neutralizing B cells over the irrelevant binders that could be expanded with subsequent boosts using different Env forms. Computational methods could be used to design Env sequence and glycan modifications to decrease strain specific glycan holes with concomitant exposure of conserved sites, such as the CD4bs, that will have a chance to promote the development of neutralization breadth. Finally, driving robust germinal center persistence through novel delivery methods and adjuvants may also provide key advances towards eliciting durable and broadly protective antibody responses.

## Materials and methods

### Ethics statement

The previous nonhuman primate study and all procedures were reviewed and approved by the Emory University Institutional Animal Care and Use Committee (IACUC) and complied with NIH guidelines. The previous animal research was also in compliance with the Animal Welfare Act and other Federal statutes and regulations relating to experiments involving animals. All animal research adhered to the principles stated in the 2011 Guide for the Care and Use of Laboratory Animals prepared by the National Research Council. The Emory National Primate Research Center (ENPRC) is fully accredited by the Association for Assessment and Accreditation of Laboratory Animal Care (AAALAC). Methods of euthanasia were consistent with the American Veterinary Medical Association with Guidelines.

### Animals and immunizations

This study has been described in detail [11]. Briefly, 20 Indian origin rhesus macaques (*Macaca mulatta*), including 17 male and 3 female, were immunized intramuscularly as follows: week 0 and week 8 with pGA1-SHIV-1-R66M/Z1800M DNA (3 mg each time point); week 16 and week 24 with rMVA-SHIV-1-R66M/Z1800M (1 x 10^8^ PFU each time point); weeks 53 and 61 with either gp120 or trimer protein (100 μg each time point). The RM were split into 2 groups of 10 animals each and one group received reagents derived from the HIV-1 T/F Env Z1800M E4.02 sequence while the other received reagents derived from the HIV-1 T/F Env R66M O20.03 sequence [13]. For the protein immunizations, the groups of 10 were further subdivided with 5 animals receiving gp120 protein and the other 5 animals receiving the stabilized gp140 trimer. For serum collection, whole blood was collected into SST tubes and spun at 4°C for 30 min. at 2000 rpm in a tabletop centrifuge. Separated serum was heat inactivated at 56°C for 45 min., aliquoted and stored at -80°C. For PBMC isolation, whole blood was collected into CPT tubes (Becton Dickinson (BD) Biosciences vacutainer catalog number 362761), processed according to the manufacturer’s recommendations and purified PBMC were cryopreserved in 90% FBS/10% DMSO in liquid nitrogen vapor phase storage.

### Flow cytometric staining of antigen specific memory B cells for 10X Genomics sequencing

scRNAseq was performed on antigen specific, IgG+ circulating B cells from PBMC cryopreserved at weeks 10, 26, 55, 63, and 90 (necropsy). Sorts were performed using four samples (animal/time point) at a time. The number of sorted antigen specific cells per sample ranged from 12 to 18,023 and the number sequenced per sample ranged from 0 to 7,520. The gating strategy for sorting was size, singlets, live, CD14-, CD3-, CD20+, IgG+, and gp120-His double positive. Each vial of cryopreserved PBMC containing 5–10 x 10^6^ cells was washed and resuspended in sterile filtered PBS + 5% FBS. Cells were counted and 5–10 x 10^6^ PBMC were incubated with either Z1800M gp120-His or R66M gp120-His for 15 min. at room temp. or on ice, then stained in a 100 μl volume with live/dead dye Fixable Viability Dye eFluor780 (catalog number 65-0865-14, ThermoFisher Scientific eBioscience), anti-human CD14 PE-Cy7 (catalog number 367111, BioLegend), anti-human CD3 Pacific Blue (catalog number 317313, BioLegend), anti-human CD20 BV650 (catalog number 302355, BioLegend), anti-human IgG FITC (catalog number 555786, BD Pharmingen), anti-His PE (catalog number 130-120-718, Miltenyi Biotec), anti-His APC (catalog number 130-119-782, Miltenyi Biotec) for 30 min. at 4°C in the dark. A cell hashing antibody that was unique for each animal was included in the phenotyping panel, so that sorted cells could be pooled for 10X Genomics sequencing and later deconvoluted by hashtag barcode. The following hashtags were used at 4 μl per 5 x 10^6^ cells: TotalSeq-C anti-human Hashtag 1 (catalog number 394661, Biolegend), TotalSeq-C anti-human Hashtag 2 (catalog number 394663, Biolegend), TotalSeq-C anti-human Hashtag 3 (catalog number 394665, Biolegend), TotalSeq-C anti-human Hashtag 4 (catalog number 394667, Biolegend,), TotalSeq-C anti-human Hashtag 5 (catalog number 394669, Biolegend), TotalSeq-C anti-human HLA-DR (catalog number 307663, Biolegend), TotalSeq-C anti-human CD22 catalog number 363516, Biolegend), TotalSeq-C anti-human HLA A/B/C (catalog number 311449, Biolegend), TotalSeq-C anti-human CD40 (catalog number 334348, Biolegend). After staining, cells were washed twice with cold FACS buffer by centrifugation at 300xg for 5 min. and resuspended in FACS buffer. The sort was performed on a BD FACS Aria-II into 1.7 ml Eppendorf tubes that were precoated with FBS and contained 100 μl of R10 media (RPMI with 10% FBS).

### 10X Genomics RNA sequencing of VDJ and VJ regions

Freshly sorted cells were partitioned into droplets (Gel Bead-in-Emulsions, GEMs) using the Chromium Single Cell 5’ Library/Gel Bead kits on the 10X Chromium Controller. GEM-reverse transcriptions products were progressed through cDNA amplification and then 5’ gene expression library construction using Ig target enrichment for VH and VL chain V(D)J transcripts using a set of in-house primers designed for RM. Ig enriched libraries underwent cDNA amplification via V(D)J Enriched Library Kits. Libraries were stored at -20°C until sequencing. Libraries for 5’ transcriptomes and B cell receptor sequences were sequenced as paired end 26x91 or 100x100 nucleotide reads on an Illumina NovaSeq 6000 device. Sequencing depth was targeted to 10^5^ reads per cell. CellRanger (v) mkfastq was used to convert the raw base call (BCL) files to FASTQ files. The FASTQ files were then aligned to mmul10 genome as a reference. Filtering and preprocessing were done using CellRanger. The raw counts matrix was then used for downstream analysis with Seurat (v4.3.0) in R. CellRanger VDJ (10X Genomics v3.1.0) was used to identify the B cell receptor rearrangements present in the samples. Our custom RM IgG database [3,58–60] was used to annotate the B cell receptor sequences as this resulted in identification of more chains. Reads mapping to RM Ig loci were assembled and annotated using an individualized RM Ig germline database. B cell clonotypes (or lineages) were defined as having the same VH germline, JH segment, VL germline, JL segment, CDR3 length, and CDR3 amino acid identity greater than 70% for both VH and VL.

### Germline inference for an individualized database using RLk17 in B cells

To accurately assign germlines, we used a strategy described by [61] and [3] incorporating elements of PCR like that described in [62] to generate repertoire sequencing of the IgM heavy chains. Using PBMCs in RLT Buffer, RNA was isolated using the Qiagen RNeasy Mini kit and quality was assessed on the Agilent Bioanalyzer. Reverse transcription was performed using Clontech SMARTer II cDNA template switching, which incorporates a common 5’ primer during the template switch for full length transcript amplification. After cDNA synthesis, individual samples were amplified in three separate reactions each using a unique forward primer that contained a 5’ Illumina adaptor sequence; a 4-nt diversity randomized sequence; a 6 nucleotide-Illumina barcode; and a 3’ anchor sequence specific to unique regions in the VH1, VH3 and VH4 leader region. A common reverse primer was used for all reactions that was comprised of a 3’ anchor sequence to the 5’ end of the IgM constant region. Amplicon libraries were sequenced on an Illumina MiSeq as 309 paired end runs targeting >10 reads per cell. The sequences of germline alleles were inferred using the individual database by means of the IgDiscover algorithm [16]. IgDiscover only produced V gene alleles for the individualized database generated for RLk17. We therefore combined the individualized database and a database from Cirelli *et al*. [3], which contains V, D, and J alleles, to annotate the VH chains of the neutralizing lineages. The alleles identified for all RM are provided in S1–S20 Files.

### Isolation of plasmablasts from blood

PB were isolated according to [63]. Briefly, fresh blood collected in CPT tubes at 4 days post-immunization was centrifuged at 2,820 rpm for 30 min. with no brake at room temp. Plasma above the lymphocyte layer was discarded and lymphocytes/monocytes were recovered and transferred to a 15 ml conical tube. The CPT tube was washed with 1 ml PBS between the gel barrier and the tube wall, and this was transferred into the 15 ml conical tube to increase recovery. The cells were then pelleted by centrifugation at 1,500 rpm for 10 min. at 4°C, the supernatant was aspirated, and the pellet was loosened by flicking the tube. 3 to 4 ml of ACK lysing buffer (Ammonium, Chloride, Potassium; catalog number A1049201, ThermoFisher Scientific) was added slowly to the cell pellet, which was resuspended by pipetting gently up and down. The resuspended cells were incubated for 3–5 min. at room temp. with mixing every 1 min. The cell suspension was then transferred to a new 15 ml conical tube and 4 ml of P2 buffer (PBS, 2% FBS, sterile filtered), was added. The cells were pelleted by centrifugation at 1,200 rpm for 10 min., resuspended in 4 ml of P2, and pelleted again. The pellet was loosened and resuspended in the antibody master mix consisting of a total volume of 750 μl and then incubated for 10 min. at 4°C. The staining panel was as follows: 50 μl of anti-human CD3 AF700 (catalog number 557917, BD Pharmingen), 50 μl of anti-human CD16 AF700 (catalog number 560713, BioLegend), 100 μl of anti-human CD20 V450 (catalog number 561164, BD Horizon), 50 μl of anti-human HLA-DRPE-Texas Red (catalog number MHLDR17, Invitrogen), 100 μl of anti-human CD14 PE-Cy7 (catalog number 557742, BD Pharmingen), 50 μl of anti-human CD11c APC (catalog number 301614, BioLegend), 100 μl of anti-human CD123 PerCP-Cy5.5 (catalog number 558714, BD Pharmingen), 250 μl of anti-human CD80 PE (catalog number 557227, BD Pharmingen). After staining, cells were pelleted at 1,500 rpm for 10 min. and resuspended in 500 μl of P2 buffer. Cells were sorted immediately using a BD FACS Aria II into 2 ml tubes containing 1 ml cold RPMI media and centrifuged at 600xg for 10 min. at 4°C. Then, 900 μl of supernatant was discarded and 350 μl of Buffer RLT (catalog number 79216, Qiagen) was added, followed by mixing with a vortex and storage at -80°C for future processing.

### Immunoglobulin repertoire sequencing in plasmablasts

To amplify and sequence the VDJ region from sorted PB, the same strategy for assigning germlines described above was used with the following changes. RNA was extracted using the Qiagen RNeasy Micro kit. Instead of amplifying the IgM constant region, the IgG constant region was amplified. There were two technical replicates for each RM. We down sampled the raw data to 150,000 reads and used the sequences that were common between the replicates. Analysis of this data was performed using Immcantation (v4.1.0). Paired raw reads were assembled using PRESTo. Low quality assembled reads with a PHRED score of less than 20 were removed. V-region primers were masked to maintain the length of VDJ sequences. Duplicate sequences were collapsed and subsetted to chains with at least two representatives. These chains were then annotated using the custom database as described in the results. The functional chains were filtered to remove any without a defined CDR3 region. This set of functional chains was used for downstream clonal analysis using the same parameters for defining clones (same VH germline, JH segment, VL germline, JL segment, CDR3 length, and CDR3 amino acid identity greater than 70% for both VH and VL).

### Isolation of mAbs from immunized rhesus macaque RLk17

Methods, isolation, and characterization of the neutralizing mAbs V5.1B1, V5.1B3, V5.1C4, V5.1C10, V5.1D7, V5.1E9, V5.2A1, V5.2D10, V5.2D11, 2D6, 2B8, 2G5, 2D1, V5.1A10, and 2A10 from single, antigen-specific B cells were described previously [11,12,15]. Briefly, week 63 cryopreserved PBMC from RLk17 were washed and resuspended in PBS + 5% FBS. To select for B cells that produced neutralizing antibodies, PBMC were incubated with a Z1800M T/F Env gp120-AF647 protein containing a V5 deletion at E447 (HXB2 460) and with the wildtype Z1800M T/F gp120-His (labeled with anti-His-PE) and sorted following the approach described above. This strategy selected for cells positive for Z1800M T/F gp120-His binding and negative for binding to Z1800M gp120 with D11 mutated V5-AF647. Sorts were carried out using a BD FACS Aria II with the following gating strategy: size, singlets, live, CD14-, CD3-, CD20+, IgG+, gp120/His+/AF647- into 96-well plates containing 10 μl cell lysis buffer (catalog number 4458235, Invitrogen) and RNaseOUT (catalog number 10777019, Invitrogen). Plates were temporarily placed on dry ice before moving to -80°C for storage. The VDJ and VJ amplicons from single B cells were combined with human IgG VH and VL constant regions for expression using methods described in [11,12,15,64].

### mAbs synthesized from 10X Genomics derived VDJ sequences

100 μg each of mAbs 22UW4-1 through 22UW4-10 were produced by Sino Biological via combination of the VDJ regions from 10X Genomics scRNAseq with human IgG1 constant regions. mAbs were transiently expressed in HEK293 cells and purified by Protein A affinity chromatography. mAbs were formulated in PBS pH 7.4, filtered through a 0.2 μm filter, and stored at -80°C. Concentrations were measured by absorption at UV_280_.

### Neutralization assay

Neutralization was measured using serially diluted, heat-inactivated immunized RM serum, or mAbs in the TZM-bl assay as previously described, using cells seeded one day prior to the assay [8,13,15,65–75]. In brief, Env PV was generated by transfecting the Env-expressing plasmid DNA alongside the HIV-1 SG3ΔEnv proviral backbone DNA into 293T cells, using the Fugene HD reagent as recommended (catalog number E2311, Promega). PV stocks were collected from the 293T cell supernatants at 48 hours post transfection, clarified by centrifugation, aliquoted into small volumes, and frozen at –80°C. 2,000 IU of each titered Env PV in DMEM with 3.5–6% (vol/vol) FBS (catalog number SH30070.03, Hyclone Defined Fetal Bovine Serum, Cytiva) and 40 μg/ml DEAE-dextran was mixed with five-fold serial dilutions of heat-inactivated immunized RM serum, or mAb, and assayed for inhibition at 48 hours post-infection by lysing the cells and measuring luciferase activity using the Agilent/BioTek Cytation3 multimode microplate reader. The average background luminescence from a series of uninfected wells was subtracted from each experimental well. Experimental wells were compared against virus without a test reagent (100% infectivity). All assays contained duplicate wells and were repeated at least once independently. Neutralization ID_50_ or IC_50_ titer values were calculated in Graphpad Prism using the dose–response inhibition analysis function with variable slope, log-transformed x values, and normalized y values.

### Statistics

All statistics were calculated in R. For comparison of non-parametric samples, the Wilcoxon signed rank test was used. A p-value of < 0.05 was considered significant.

## Supporting information

S1 Fig Neutralization associated germline allele from RLk17 was not present in other Z1800M Env immunized RM. The germline VH allele associated with the development of neutralizing antibodies in RLk17 was present in 4 other RMs, all immunized with the R66M Env containing vaccines. In animals that did not harbor this germline allele, the allele with the highest identity is shown. One animal that received the Z1800M trimer vaccine, RYf17, had a highly similar germline but did not develop neutralizing antibodies.
(TIF)

S2 Fig Clonal expansion in antigen specific B cells during immunizations in all animals. Circos plots illustrating the clonal overlap and recall of antigen specific MBC throughout immunization for all animals for which sufficient cells were captured and sequenced. Ribbons are color coded as follows: second DNA = red, second MVA = blue, first protein = green, second protein = yellow. RLk17 is the only animal that developed persistent neutralizing antibodies. The color of each ribbon indicates the immunization time point at which the clonotype was first identified.
(TIF)

S1 File Individualized Ig V gene allele databases for 6S0. The sequences of germline alleles were inferred using the individual database by means of the IgDiscover algorithm [16] and a database from [3].
(TXT)

S2 File Individualized Ig V gene allele databases for 15C172. The sequences of germline alleles were inferred using the individual database by means of the IgDiscover algorithm [16] and a database from [3].
(TXT)

S3 File Individualized Ig V gene allele databases for MA277. The sequences of germline alleles were inferred using the individual database by means of the IgDiscover algorithm [16] and a database from [3].
(TXT)

S4 File Individualized Ig V gene allele databases for RFj17. The sequences of germline alleles were inferred using the individual database by means of the IgDiscover algorithm [16] and a database from [3].
(TXT)

S5 File Individualized Ig V gene allele databases for RGi17. The sequences of germline alleles were inferred using the individual database by means of the IgDiscover algorithm [16] and a database from [3].
(TXT)

S6 File Individualized Ig V gene allele databases for RLf17. The sequences of germline alleles were inferred using the individual database by means of the IgDiscover algorithm [16] and a database from [3].
(TXT)

S7 File Individualized Ig V gene allele databases for RLk17. The sequences of germline alleles were inferred using the individual database by means of the IgDiscover algorithm [16] and a database from [3].
(TXT)

S8 File Individualized Ig V gene allele databases for RLl17. The sequences of germline alleles were inferred using the individual database by means of the IgDiscover algorithm [16] and a database from [3].
(TXT)

S9 File Individualized Ig V gene allele databases for ROa17. The sequences of germline alleles were inferred using the individual database by means of the IgDiscover algorithm [16] and a database from [3].
(TXT)

S10 File Individualized Ig V gene allele databases for RPh17. The sequences of germline alleles were inferred using the individual database by means of the IgDiscover algorithm [16] and a database from [3].
(TXT)

S11 File Individualized Ig V gene allele databases for RPz16. The sequences of germline alleles were inferred using the individual database by means of the IgDiscover algorithm [16] and a database from [3].
(TXT)

S12 File Individualized Ig V gene allele databases for RRb17. The sequences of germline alleles were inferred using the individual database by means of the IgDiscover algorithm [16] and a database from [3].
(TXT)

S13 File Individualized Ig V gene allele databases for RSb17. The sequences of germline alleles were inferred using the individual database by means of the IgDiscover algorithm [16] and a database from [3].
(TXT)

S14 File Individualized Ig V gene allele databases for RTe17. The sequences of germline alleles were inferred using the individual database by means of the IgDiscover algorithm [16] and a database from [3].
(TXT)

S15 File Individualized Ig V gene allele databases for RTm17. The sequences of germline alleles were inferred using the individual database by means of the IgDiscover algorithm [16] and a database from [3].
(TXT)

S16 File Individualized Ig V gene allele databases for RVe17. The sequences of germline alleles were inferred using the individual database by means of the IgDiscover algorithm [16] and a database from [3].
(TXT)

S17 File Individualized Ig V gene allele databases for RVh17. The sequences of germline alleles were inferred using the individual database by means of the IgDiscover algorithm [16] and a database from [3].
(TXT)

S18 File Individualized Ig V gene allele databases for RVn17. The sequences of germline alleles were inferred using the individual database by means of the IgDiscover algorithm [16] and a database from [3].
(TXT)

S19 File Individualized Ig V gene allele databases for RWb17. The sequences of germline alleles were inferred using the individual database by means of the IgDiscover algorithm [16] and a database from [3].
(TXT)

S20 File Individualized Ig V gene allele databases for RYf17. The sequences of germline alleles were inferred using the individual database by means of the IgDiscover algorithm [16] and a database from [3].
(TXT)
