# Supplementary figures and images for: High throughput analysis of B cell dynamics and neutralizing antibody development during immunization with a novel clade C HIV-1 envelope
Source: PLoS Pathog. 2023 Oct 25;19(10):e1011717. doi: 10.1371/journal.ppat.1011717 (PMC10627474; doi:10.1371/journal.ppat.1011717)

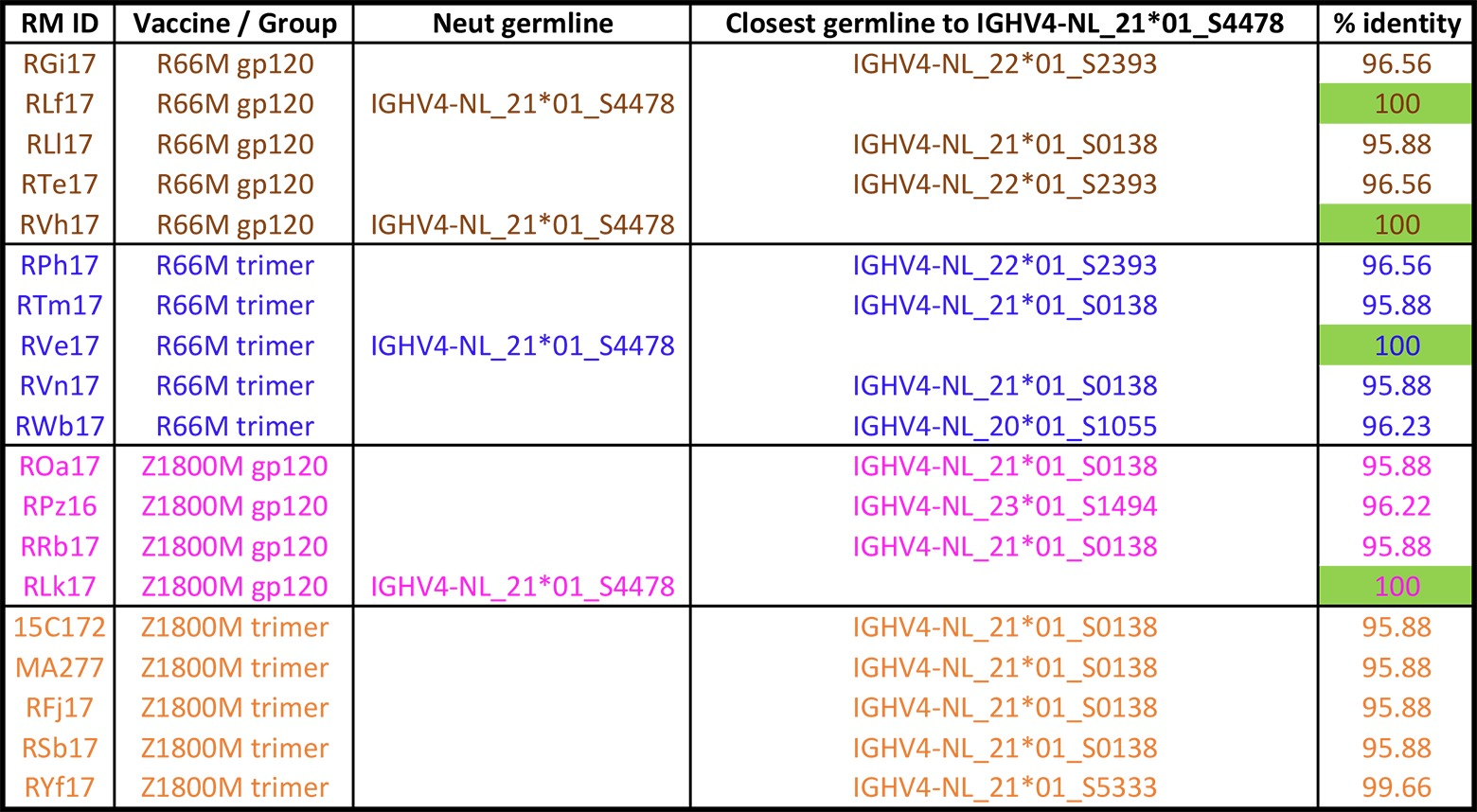

Supplement: S1 Fig — The germline VH allele associated with the development of neutralizing antibodies in RLk17 was present in 4 other RMs, all immunized with the R66M Env containing vaccines. In animals that did not harbor this germline allele, the allele with the highest identity is shown. One animal that received the Z1800M trimer vaccine, RYf17, had a highly similar germline but did not develop neutralizing antibodies. (TIF) [file ppat.1011717.s001.tif]

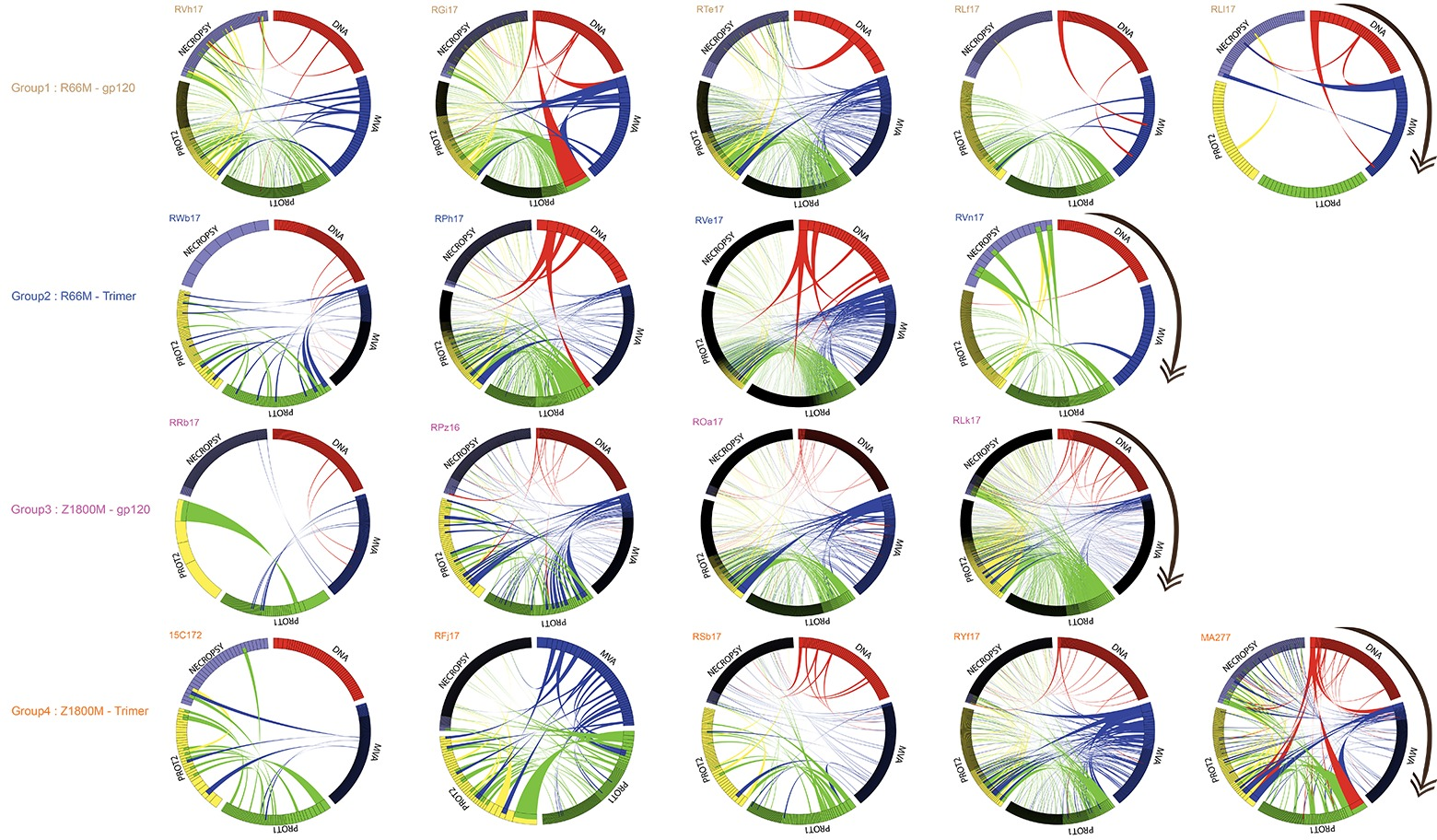

Supplement: S2 Fig — Circos plots illustrating the clonal overlap and recall of antigen specific MBC throughout immunization for all animals for which sufficient cells were captured and sequenced. Ribbons are color coded as follows: second DNA = red, second MVA = blue, first protein = green, second protein = yellow. RLk17 is the only animal that developed persistent neutralizing antibodies. The color of each ribbon indicates the immunization time point at which the clonotype was first identified. (TIF) [file ppat.1011717.s002.tif]
